# Long-Term Response of Groundwater Nitrate Concentrations to Management Regulations in Nebraska's Central Platte Valley

**DOI:** 10.1100/tsw.2010.25

**Published:** 2010-02-17

**Authors:** Mary E. Exner, Hugo Perea-Estrada, Roy F. Spalding

**Affiliations:** ^1^ School of Natural Resources, Institute of Agriculture and Natural Resources, University of Nebraska, Lincoln, USA; ^2^ Department of Agronomy and Horticulture, Institute of Agriculture and Natural Resources, University of Nebraska, Lincoln, USA

**Keywords:** groundwater nitrate, water management, N management, nitrogen management, N-use efficiency, Nebraska, irrigation, groundwater quality, groundwater policy, groundwater quality regulations

## Abstract

The impact of 16 years (1988–2003) of management practices on high groundwater nitrate concentrations in Nebraska's central Platte River valley was assessed in a 58,812-ha (145,215-ac) groundwater quality management area intensively cropped to irrigated corn (*Zea mays* L.). Crop production and groundwater nitrate data were obtained from ~23,800 producer reports. The terrace, comprising ~56% of the study area, is much more intensively cropped to irrigated corn than the bottomland. From 1987 to 2003, average groundwater nitrate concentrations in the primary aquifer beneath the bottomland remained static at ~8 mg N/l. During the same period, average groundwater nitrate concentrations in the primary aquifer beneath the terrace decreased from 26.4 to 22.0 mg N/l at a slow, but significant (*p* < 0.0001), rate of 0.26 mg N/l/year. Approximately 20% of the decrease in nitrate concentrations can be attributed to increases in the amount of N removed from fields as a consequence of small annual increases in yield. During the study, producers converted ~15% of the ~28,300 furrow-irrigated terrace hectares (~69,800 ac) to sprinkler irrigation. The conversion is associated with about an additional 50% of the decline in the nitrate concentration, and demonstrates the importance of both improved water and N management. Average N fertilizer application rates on the terrace were essentially unchanged during the study. The data indicate that groundwater nitrate concentrations have responded to improved management practices instituted by the Central Platte Natural Resources District.

### INTRODUCTION

Limiting nitrate leaching from cropland to groundwater is imperative, as nitrate is a serious human health hazard in drinking water and an ecological threat in groundwater discharge. The U.S. Environmental Protection Agency has set 10 mg N/l as the Maximum Contaminant Level (MCL) for nitrate in drinking water[1], as consumption of high-nitrate water can cause methemoglobinemia, a potentially fatal condition in infants[2]. In the U.S., groundwater is the source of 37% of the public-supply water and of 98% of the potable water used by the 15% of the population that is self-sufficient[3]. When discharged to surface water, excess nitrate is associated with the proliferation of the toxic blue-green algae *Microcystis*[4] and with hypoxia. Hypoxic zones like that in the northern Gulf of Mexico damage the marine ecosystem and adversely impact commercial and recreational fisheries.

Nitrate contamination of groundwater has been linked to nitrate leaching from cropland in the major row-cropped regions of the U.S. Areas with high inputs of agricultural N and high aquifer vulnerability have increased risk of groundwater nitrate contamination. Areas of highest risk occur in the western, midwestern, and northeastern U.S.[5]. Both N and water inputs are higher on irrigated than nonirrigated cropland; consequently, shallow aquifers beneath irrigated cropland are most vulnerable to nitrate contamination[6].

In Nebraska, 85% of the population relies on groundwater for their potable water. An assessment of 5,800 wells[6] sampled between 1984 and 1989 found that 20% of the wells exceeded the 10 mg N/l MCL. More than half of the wells exceeding the MCL were located in intensively row-cropped areas characterized by fence-row to fence-row, irrigated, predominantly corn monoagriculture, well-drained soils, and a short distance(<15 m [<50 ft]) to groundwater. The most extensive contamination occurred in the 8,547-$km^2$ (3,300-$mi^2$) Central Platte Natural Resources District (CPNRD), which comprises the Platte River watershed on the north side of the Platte River in central Nebraska (Fig. 1). Historically, average nitrate-N concentrations in the CPNRD's three eastern counties of Merrick, Hall, and Buffalo (Fig. 1) increased between 1961 and 1984[7,8,9,10]. Stable N isotope studies confirmed that leachate from the application of commercial N fertilizer was the major source of the nitrate contamination[11].

Natural Resources Districts (NRDs) are local governmental entities created in 1972 by Nebraska statute and charged with the development, management, utilization, and conservation of groundwater and pollution control[12]. The Groundwater Management and Protection Act of 1981 granted NRDs authority to protect groundwater quality; however, nonpoint source contamination was not seriously addressed until sweeping revisions to the Act in 1986 authorized NRDs to form management areas, primarily to protect groundwater quality, and allowed NRDs to require implementation of best management practices and attendance at educational programs designed to protect water quality[13]. These legislative actions led the CPNRD to develop Nebraska's first groundwater quality management plan (GWQMP). Implemented in 1987, the plan has become a model for NRDs confronted with nonpoint source groundwater nitrate contamination.

The CPNRD's GWQMP uses a phased approach to regulation. Areal nitrate concentrations in irrigation wells are averaged to delineate three management phases. Presently, areas with nitrate concentrations averaging ≤7.5, 7.6–15, and ≥15.1 mg N/l are designated Phase I, II, and III, respectively. Each phase has increasingly restrictive management rules and regulations. With the implementation of the GWQMP in 1987, 58,812 ha (145,215 ac) in eastern Buffalo and western Hall Counties were designated as Phase II, and in 1992 became a Phase III management area (Fig. 1). Within the area, the timing and application rates of N fertilizer on irrigated corn, grain sorghum (*Sorghum bicolor* L.[Moench]), and potatoes (*Solanum tuberosum* L.) are regulated. Beginning with the 1988 crop season, annual reports detailing many variables, including N credits in soil and irrigation water, and N fertilizer recommendation and application rates, were required for each irrigation well and its acreage of regulated crop. The objectives are to utilize this rich dataset in order to determine groundwater nitrate concentration trends in the primary aquifer, and to evaluate the impact of N inputs and N removal on groundwater nitrate concentrations in the primary aquifer at this U.S. Department of Agriculture's Cooperative State Research, Education and Extension Service (USDA-CSREES) Conservation Effects Assessment Project (CEAP) study site.

The goal of the USDA-CSREES's CEAP, a joint effort with the USDA Natural Resources Conservation Service (NRCS) and Farm Service Agency (FSA), is to determine the effectiveness of agricultural conservation practices measured at the field or plot scale on the actual restoration of chemical, physical, and/or biological water quality at the watershed scale. There are 13 case studies at already-intensively-studied watersheds throughout the U.S. Each is supported by a large existing database, a precondition of funding. The results presented here are for the only case study assessing agricultural conservation practices on groundwater quality.

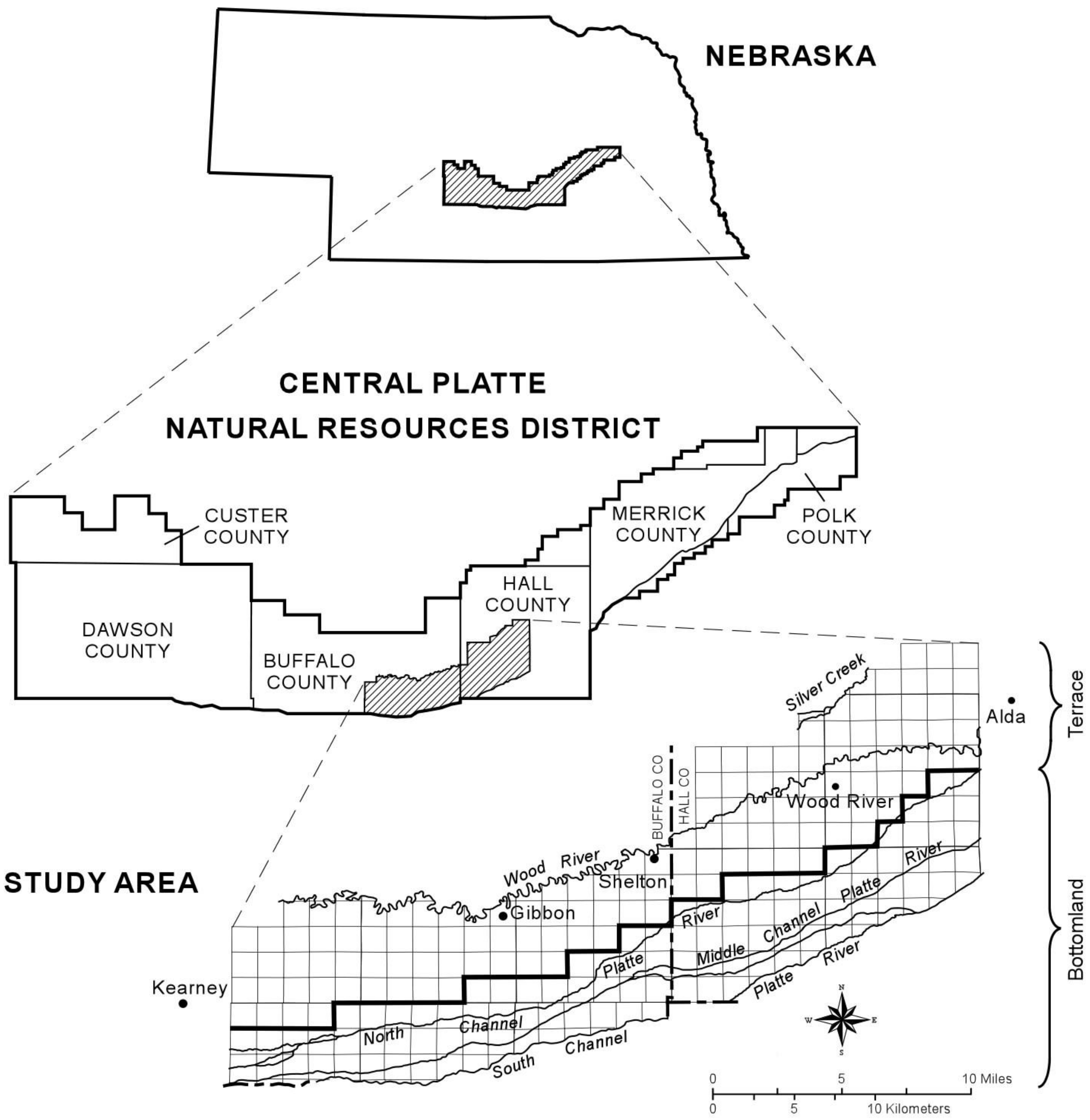

**FIGURE 1.** CPNRD Phase III groundwater quality management area in Buffalo and Hall Counties of Nebraska. The bold black line in the bottom panel represents the topographical divide between the bottomland and terrace.

### MATERIALS AND METHODS

#### Study Site

The 58,812-ha (145,215-ac) study site is located in the Platte River valley of south-central Nebraska (Fig. 1) and is in the Wood hydrologic unit (HUC 10200102). The site is about 47 km (29 mi) long and averages about 6 km (8 mi) in width. The valley has very little relief; consequently, runoff is minimal. The terrace is 1.5–3 m (5–10 ft) above a broad bottomland[14] and is separated from the bottomland by an obvious gradient that parallels the North Channel of the Platte River. Groundwater beneath the study area is easily accessible and abundant, and is the sole source of irrigation water. Differences in soil drainage, land use, and hydrogeology distinguish the terrace and bottomland.

On the terrace, about 1–2 m (4–6 ft) of well-drained, silty loam soils and medium-to-fine sands overlie the saturated zone, which lies about 1.5–7.5 m (5–25 ft) below the land surface. Three distinct hydrogeologic units comprise the saturated zone: a shallow primary aquifer, an aquitard, and a secondary aquifer. About 10.5–17 m (35–55 ft) of Pleistocene sand and gravel comprise the primary aquifer. The aquifer's lower boundary is a 9- to 20-m (30- to 65-ft)-thick aquitard comprised of clayey silt interbedded with clay zones and sand. The secondary aquifer below the aquitard has a very high level of heterogeneity. The secondary aquifer is absent in the eastern part of the study site; however, its thickness increases to the west, reaching 46 m (150 ft) near Kearney. Composed of lime-cemented sandstone, it is much less permeable than the overlying sand and gravel primary aquifer.

The terrace of the study site nearly coincides with the high nitrate (≥10 mg N/l) concentration areas previously delineated in the primary aquifer beneath the terrace of eastern Buffalo and western Hall Counties in 1974[9]. A follow-up study in 1984 revealed that in the intervening decade, concentrations in the high nitrate zones had increased at average annual rates of 0.8–1 mg N/l, that the areal extent of the contamination had increased, and that high nitrate zones had merged and averaged 22 ± 8 mg N/l[10].

Approximately 44% of the study site is Platte River bottomland. Poorly to excessively drained soils grade to mixed sands and gravels above the primary aquifer. The water table is 1–2 m (3–6 ft) below the land surface. In the study area, the Platte River is a losing stream and a source of very low nitrate (<1 mg N/l) groundwater.

The terrace is intensively cropped and ~86% is irrigated crop land. Each year between 1988 and 2003, ~80% of the irrigated crop land was dedicated to corn production. Prior to 1993, seed corn production constituted <1% of the acres planted to corn. The acreage gradually increased to ~5% in 1997 and remained stable until 2003, when it decreased to ~3%. The reliability of the seed corn production data was questionable and those data are not included in this discussion.

About 67% of the bottomland is irrigated. During the study, ~45% of the irrigated area was cropped to corn. Seed corn production was negligible.

Furrow irrigation is the predominant irrigation method on both the terrace and bottomland.

### Regulations

In the study area, producers of regulated crops (irrigated corn, grain sorghum, and potatoes) must adhere to rules and regulations set by the CPNRD. Sorghum and potato acres were negligible with ~400 ha (1,000 ac) planted to these crops during the entire study period. The regulations address the timing and application of N fertilizer, the content of a two-part annual report submitted to the CPNRD, monitoring of the irrigation water application, and operator certification and education. Annual reports must be prepared for each well that irrigates a regulated crop with the field identified by the well's location. A maximum of ~65 ha (160 ac) can be included in one report as long as all the land is irrigated by the same well and planted to the same crop. The following regulations were in effect between 1987 and 2003 with the only significant change affecting the timing and application of commercial N fertilizer. Before 1992, fall application of commercial N fertilizer was not permitted on sandy soil, but application with the use of an approved inhibitor was permitted on heavier soils after November 1. Since 1992, fall and winter commercial N fertilizer applications have been banned on all soils. Spring applications are banned until after March 1, with applications of >90 kg N/ha (80 lb N/ac) either split between pre-emergence and side-dress, or applied with an approved inhibitor and dealer certification of its use at the recommended rate. Nitrogen in soils and irrigation water, in manure applications, and from a previous legume crop must be credited in determining the CPNRD fertilizer recommendation. Nitrate-N in soils and irrigation water must be measured annually and laboratory reports submitted to the CPNRD. The minimum soil sampling requirement is one composite sample of 16 0.9-m (3-ft) probes for every 32 ha (80 ac), although one composite of eight probes for every 16 ha (40 ac) is recommended. One water sample from the irrigation well is obtained during the previous irrigation season to determine the N credit in the recommended application of 229 mm (9 in) of irrigation water. While manure applications must be credited and the results of a laboratory analysis for each manure source are required, reports of manure applications were almost nonexistent in the study area. Credits for the previous season's legume crop are 50 and 134 kg N/ha (45 and 120 lb N/ac) for soybeans and alfalfa (*Medicago sativa* L.), respectively. These credits, the expected crop yield goal (105% of the previous 5-year average yield), and the N rate needed to attain the expected yield are used to calculate the CPNRD's recommended N application rate. Additional data reporting requirements include actual yield, pre-emergence, postemergence, and total N application rates, and amount of irrigation water applied.

### Sampling and Analytical Methods

Soil and water samples were analyzed by commercial laboratories located in the nearby towns of Hastings, Kearney, and Gibbon. The samples normally were collected and brought to the laboratory by crop consultants. All laboratories used the same extraction and analytical procedures. Aliquots of soil nitrate were extracted with 2 *M* KCl and analyzed using the automated cadmium reduction method[15]. Irrigation well samples also were analyzed by automated cadmium reduction. Crop consultants and farmers were instructed to pump irrigation wells for at least 2 h prior to sampling[16]. The irrigation wells are all high-capacity wells and pump ~3.8 $m^3$/min (1,000 gal/min). Irrigation wells have clearly been shown to be valuable and reliable in monitoring average nitrate-N and pesticide variability in nonpoint-source contaminated areas[17].

### Data Management

The CPNRD provided each year of producer data as an Access file with fields for each of the reporting requirements and each report identified by the location of the irrigation well. These files, containing more than 23,800 reports, were combined into a producer data Access table. Registration numbers and well attribute information for the ~1,900 irrigation well locations were obtained from the Quality Assessed Agrichemical Contaminant Database for Nebraska Ground Water[18]. While much of the well attribute data in the agrichemical contaminant database was downloaded from the Nebraska Registered Groundwater Wells database[19], the well attribute data in the contaminant database include well-screen depth intervals that, for the majority of wells, were obtained from paper registrations or, for newer wells, were obtained by viewing registrations online. Well depth, screened interval(s), and the geologic log available online in the Nebraska Registered Groundwater Wells database[19] were used to determine the source aquifer(s), which were entered into the well attributes table. The well attribute and producer data tables are in an Access database and linked by the well location.

The location, year of initial use, and acres irrigated by each center-pivot irrigation system were gleaned by examining Natural Resources Conservation Service single-section (259 ha [640 ac]) aerial photo slides for each year.

## RESULTS AND DISCUSSION

Average nitrate concentrations in the primary aquifer beneath the terrace peaked at 26.8 mg N/l in 1988 (Fig. 2). Yearly average nitrate concentrations were calculated from an average of 867 data points (irrigation wells) with the number ranging from 744 in 2003, to 949 in 1990 and 1996. Groundwater nitrate concentrations in 1989 responded favorably to the first full cropping season of implemented N fertilizer management regulations (1988) and gradually declined during the study period. The average annual decline of 0.26 mg N/l was small, but highly significant ($p < 0.0001$). In 2003, at the end of the study period,

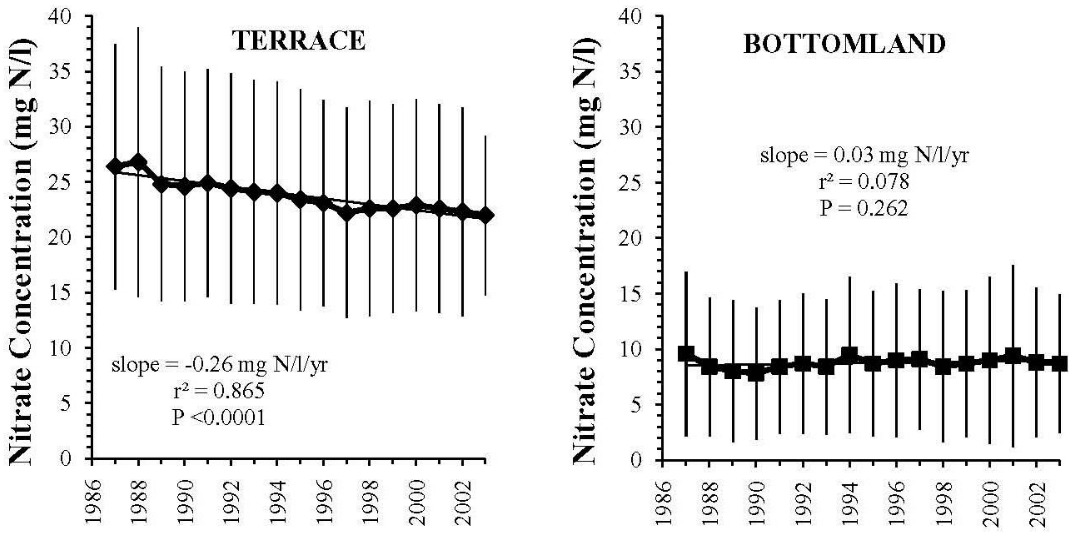

**FIGURE 2.** Groundwater nitrate concentration trends and rate of change in the primary aquifer beneath the terrace and bottomland. Vertical bars represent one standard deviation from the mean values.

concentrations averaged 22.0 mg N/l, or more than twice the MCL. This concentration was identical to the average concentration of the high nitrate zone delineated in 1984, which encompassed an area almost identical to the study area. Projecting increases of 1 mg N/l/year[10] from 1985 to 1988, the average nitrate concentration in the high nitrate zone would have reached 26 mg N/l, which compared very favorably with the peak concentration (26.8 mg N/l) attained in the study area. In a 1980–1983 study in Hall County (within the study area), corn yield goals averaged 28% above actual production, resulting in an average application of ~48 kg N/ha (~43 lb N/ac) in excess of the fertilizer recommendation[20]. In 1988, yield goals were more realistic in the groundwater management areas with mandatory regulations and reporting requirements. Corn yield goals averaged 9% more than the average actual yield of 10.0 Mg/ha (159 bu/ac), which significantly lowered the average excess N application rate to ~20 kg N/ha (~18 lb N/ac) more than the average recommendation. This reduced the N available for leaching[16].

Average nitrate concentrations in the primary aquifer beneath the bottomland were elevated and slightly below the MCL (Fig. 2). Since 1988, concentrations remained static at ~8–9 mg N/l and appear to have reached steady state. This situation likely is a consequence of two factors: irrigated corn production is much less intense in the bottomland than in the terrace, and low-nitrate Platte River seepage moves northeasterly into the bottomland of the study area and displaces the groundwater. Coincident with less irrigation is a reduction in the number of irrigation well data points used to calculate the yearly average nitrate concentrations. The number of data points ranged from a low of 169 in 1987 to 384 in 2000, and averaged 325. Because average groundwater nitrate concentrations beneath the bottomland are static and of acceptable drinking water quality, the results and discussion that follow address changes occurring in the terrace.

Between 1988 and 2003, the average N fertilizer application rate for corn grown on the terrace averaged 159 ± 12 kg N/ha (142 ± 10 lb N/ac), with only a small average annual increase of 1.0 kg N/ha (0.9 lb N/ac) (Fig. 3). Year-to-year fluctuations in N fertilizer application rates are expected and can be attributed to changes in N credits, expected yield, overwinter N losses, and volatile fertilizer prices. Noteworthy in Fig. 3 is the sharp ~25 kg N/ha (~22 lb N/ac) decline in the average N fertilizer application rate in 2001. The decrease, the largest seen during the study, followed a peak N application rate of ~177 kg N/ha (~158 lb N/ac) and appears largely the result of a near doubling in the price of anhydrous ammonia-N to $424/Mg ($385/ton)[21]. The following year, anhydrous ammonia-N prices returned to near 2000 levels[21] and, while the average N application rate rebounded, it was below the previous high. The N application rate declined again in 2003[22] as a likely consequence of another dramatic increase in

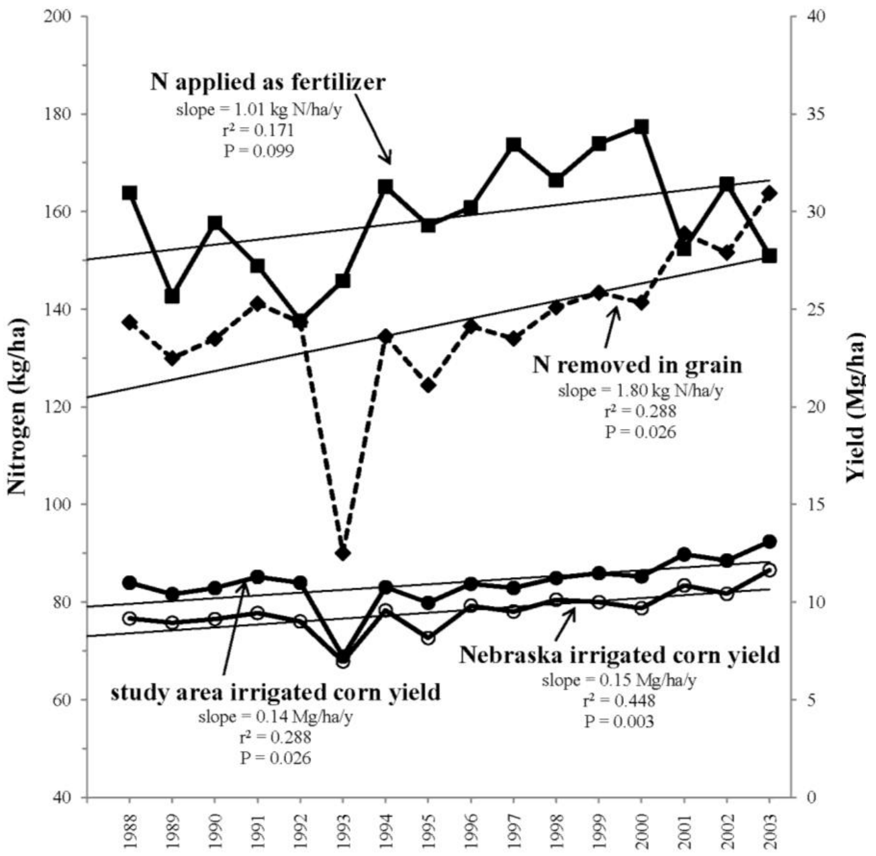

**FIGURE 3.** Commercial N fertilizer applied, N removed in grain, and grain yield for irrigated corn acres on the terrace.

fertilizer costs to near 2001 prices. N fertilizer use on the irrigated fields of the study area reflects a similarly stable pattern seen in the intensive corn production areas of the U.S. Based on their analysis of USDA Annual Cropping Practices Surveys data, Dobermann and Cassman[23] report that N fertilizer use has remained stagnant since 1980.

While average N fertilizer application rates remained essentially unchanged during the study, corn yields increased (Fig. 3) at an average rate of 0.14 Mg/ha/year (2.2 bu/ac/year). This trend of increasing corn yields while average N fertilizer application rates remained essentially unchanged has occurred throughout the Corn Belt since 1980[23]. The average increase in yield during the study is slightly lower than the 0.15 Mg/ha/year (2.4 bu/ac/year) reported for irrigated corn in Nebraska during the same period (Fig. 3). The conspicuous drop in yields in 1993 was the consequence of low photosynthetic activity due to an extremely wet, cloudy, and cool growing season. Omitting the collapse in yield in 1993 lowered, but did not substantially change, the annual increase in yield in the study area to 0.11 Mg/ha (1.8 bu/ac). Annual yields in the study area averaged 11 ± 1.3 Mg/ha (175 ± 20 bu/ac) and averaged 1.5 Mg/ha (23.8 bu/ac) greater than average yields for irrigated corn in Nebraska. The study area has long been recognized as one of the most fertile, irrigated-corn–producing areas in the Corn Belt and, for that reason, is likely to approach the yield potential ceiling[24] more quickly than other irrigated areas in Nebraska.

Large irrigated-corn yields and the 1.2% N (9 kg N/m$^3$ [0.7 lb N/bu]) content of the grain resulted in the harvest of large quantities of N from the fields (Fig. 3). During the study, average N removal rates approached average N application rates (Fig. 3). If the trend continues, N removed in grain soon will be equivalent to or surpass N application rates. Sustained N fertilizer prices at levels seen in 2001 and 2003 could accelerate the trend. In both years, more N was removed in grain than was applied as N fertilizer, with no decrease in yield. Excess N as residual soil N and in irrigation water, however, was still available for crop uptake. In the study area, only grain is removed from the field. Plant, cob, and husk remain to be recycled; some to be utilized by grazed cattle. Nitrogen in the plant material and in the cattle manure eventually are mineralized and credited in the N-budgeting process. The soil N pool in many fields in the Phase III area is considered to have reached or to be approaching quasi steady state. This is a consequence of consistent application of similar management practices on well-drained irrigated soils for many years[25].

Approximately 20% of the decrease in the nitrate concentration in the primary aquifer beneath the terrace is associated with increasing N removal rates (Fig. 4), which in turn are a consequence of increased yields. Increased yields are associated with increased resistance to stress, improved management production factors other than nutrient management, and improved N fertilizer management[26]. Pairing the average amount of N removed in the grain with the following summer's average groundwater nitrate concentration (Fig. 4) gave the best-fit line ($r^2 = 0.212$, $p = 0.084$). The 1993 N removal rate and 1994 nitrate concentration were omitted from the analysis because of uncharacteristically low yields in 1993. The quick response of the groundwater to management practices was reported at Nebraska's Management Systems Evaluation Area (NE-MSEA) located within the study area. There groundwater age-dating and vertical nitrate profiles showed that season-to-season changes in management were reflected in the shallow groundwater nitrate concentrations[27].

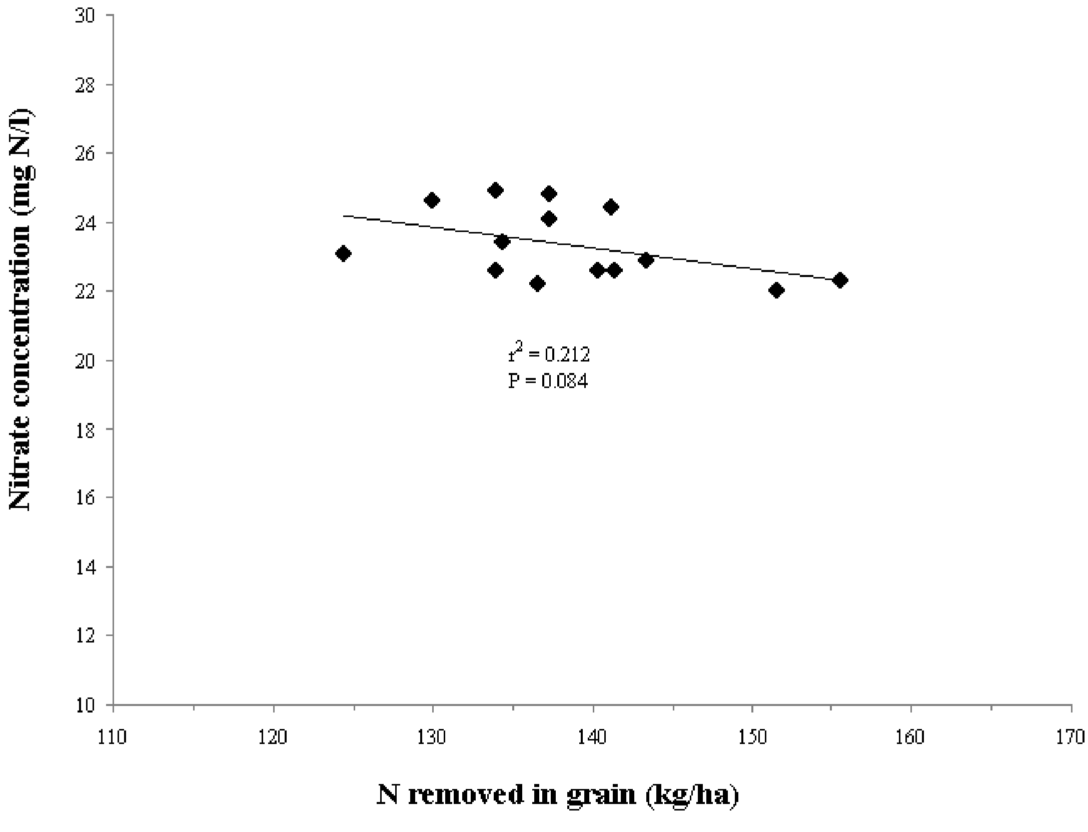

**FIGURE 4.** Relationship between groundwater nitrate concentrations in the primary aquifer beneath the terrace and N removed in grain.

Irrigation water management is critical in order to control nitrate leaching. While irrigation application rates must be reported by the producer, most data reported prior to 2000 were estimations and are highly suspect (Fig. 5). In 2000, when one-time CPNRD-measured pumping rates were completed and available for all irrigation wells, water application was calculated using a CPNRD formula. Two years later, digital consumption meters on electric wells improved the accuracy of the number of kilowatt hours used in the calculation. Irrigation application rates reported in 2000 doubled those reported in previous years. In the 4 years from 2000 to 2003, an average of 620 mm (24.4 in.) of irrigation water was applied to corn on the terrace and more accurately reflects the amount of water applied prior to 2000.

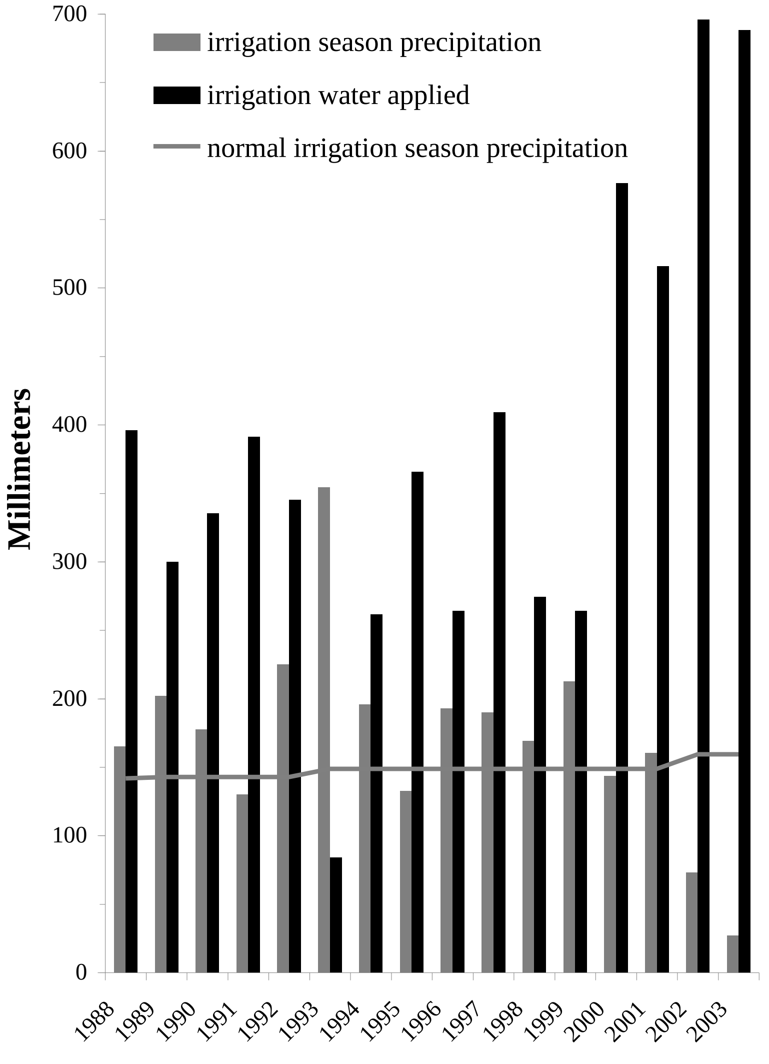

**FIGURE 5.** Irrigation season (July and August) precipitation and irrigation water application. Kearney and Grand Island station data[28] were averaged to obtain the irrigation season and normal irrigation season precipitation for the study area.

The well-drained soils and flatness of the terrace cause it to be well suited to furrow irrigation; consequently, only ~4% (1,167 ha [2,882 ac]) of the irrigated area was sprinkler irrigated in 1988. The results of the NE-MSEA's study (1991–1996) led to the conclusion that conversion from furrow to well-managed sprinkler irrigation could significantly improve shallow groundwater quality[27]. Doing so would allow more uniform water applications and timely supplemental N fertilization, both of which can limit leaching.

From 1988 through 1993, sprinkler-irrigated acres on the terrace remained constant at ~1,175 ha (~2,900 ac) or 4–4.5% of the irrigated area. Gradual increases occurred in subsequent years with the largest addition of ~1,400 ha (~3,460 ac) occurring in 2002. In that year, ~4,900 ha (12,154 ac) or ~17% of the terrace's irrigated area was under sprinkler irrigation. Since 1994, nitrate concentrations in the primary aquifer beneath the terrace appear to have benefited from the conversion from furrow to sprinkler irrigation (Fig. 6). As with the correlation between N removal rates and groundwater nitrate concentrations, the percentage of the terrace under sprinkler irrigation is paired with the following growing season's average groundwater nitrate concentration. The association is significant ($r^2 = 0.545$, $p = 0.010$) and suggests that more than half of the decrease in nitrate concentration can be attributed to the benefits of sprinkler irrigation: more uniform water applications and the ability to better synchronize N application with crop needs using chlorophyll meter readings (spoon feeding)[27,29].

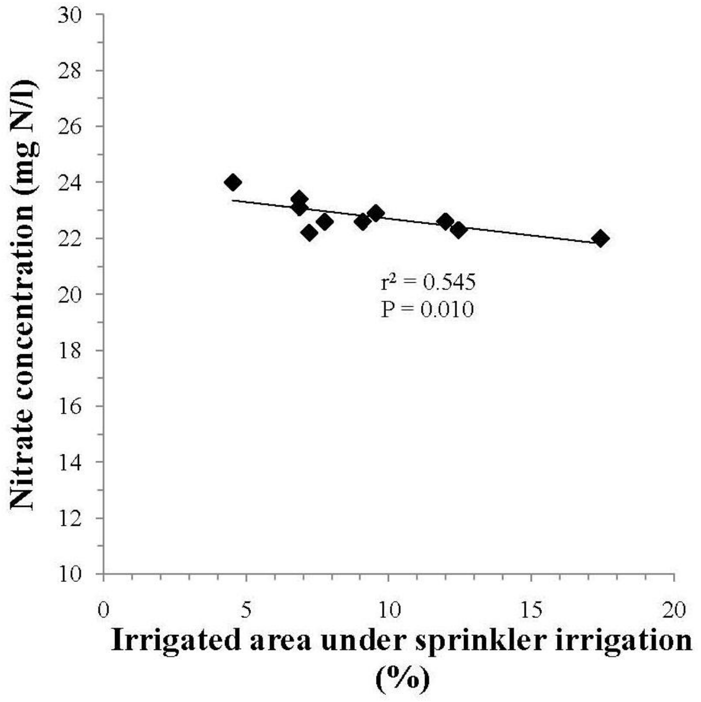

**FIGURE 6.** Relationship between groundwater nitrate concentrations in the primary aquifer and percentage of sprinkler-irrigated acres on the terrace.

Since 1998, the CPNRD has provided cost-share money to convert from furrow to sprinkler irrigation. Over the years, the incentive has increased from $3,000 to $7,500 per system. In 2009, the lifetime limitation of one system per producer was replaced by one system per producer per year with the expectation that it would overcome the slowdown in conversions. Producers accepting the incentive money agree to apply N fertilizer at the CPNRD recommended rate for 10 years. Cost-share money also is available to subsidize the cost of underground pipe associated with furrow to sprinkler conversions.

### SUMMARY AND CONCLUSIONS

The management practices instituted in the CPNRD's groundwater quality management area in 1988 have reversed the long-term trend of increasing average nitrate-N concentrations in the primary aquifer beneath the terrace. However, from the observed average rate of decline in nitrate concentrations, it will be many decades, if ever, before average nitrate levels are lowered to the 10 mg N/l MCL. Producer adoption of realistic yield goals and N budgeting as promoted by the CPNRD resulted in relatively constant average N fertilizer application rates during the period of investigation. This was an essential factor in the significant association between the increased N removal from fields as a consequence of increased grain yields and the decrease in average groundwater nitrate concentrations. About 20% of the decline in groundwater nitrate concentrations was associated with increasing N removal in grain. The increase in sprinkler-irrigated acres appears more strongly associated with the decline in average groundwater nitrate concentrations. About 50% of the decline in average nitrate levels was statistically associated with the conversion from furrow to sprinkler irrigation. The ability of center-pivot systems to apply water uniformly and to spoon feed nutrients appears to be an overriding factor in lowering groundwater nitrate levels. If producers continue to apply N using realistic yield goals, a continual increase in the number of sprinkler-irrigated acres and new incentives for converting to pivots should further benefit groundwater nitrate levels. The data indicate that in irrigated-corn production, water management and accurate water application measurements directly impact groundwater quality; thus, they are extremely important in controlling nitrate leaching.

### ACKNOWLEDGMENTS

The authors gratefully acknowledge the Central Platte Natural Resources District for providing the producer report data, and the assistance and institutional knowledge of Sandy Noecker. We are indebted to Dr. James Schepers, retired leader of Nebraska's USDA-ARS unit, for his constructive comments and critical review of the manuscript. This material is based on work supported by the Cooperative State Research, Education, and Extension Service, U.S. Department of Agriculture, under Agreement No. 2006-51130-03708.
